# Auxological and Endocrinological Features in Children and Adolescents with Cystic Fibrosis

**DOI:** 10.3390/jcm11144041

**Published:** 2022-07-13

**Authors:** Vittorio Ferrari, Vito Terlizzi, Stefano Stagi

**Affiliations:** 1 Department of Health Sciences, University of Florence, Anna Meyer Children’s University Hospital, 50100 Florence, Italy; vittorioferrari15@gmail.com; 2 Cystic Fibrosis Regional Reference Center, Department of Paediatric Medicine, Meyer Children’s Hospital, 50139 Florence, Italy; vito.terlizzi@meyer.it

**Keywords:** cystic fibrosis, *CFTR*, growth failure, short stature, puberty delay, hypothyroidism, bone, hypogonadism, infertility, diabetes, children, adolescents

## Abstract

Cystic fibrosis (CF) is a multisystem autosomal recessive disease caused by mutations that lead to deficient or dysfunctional CF transmembrane conductance regulator (*CFTR*) proteins. Patients typically present malnutrition resulting from the malabsorption of fundamental nutrients and recurring lung infections, with a progressive worsening of the respiratory function. For these reasons, the clinical management of CF requires a multidisciplinary team. From an endocrinological point of view, patients often present major complications, such as diabetes, bone disease, thyroid disorders, delayed growth and puberty, hypogonadism and infertility, which negatively affect their quality of life and, in some cases, significantly reduce life expectancy. These complications can arise as a direct result of *CFTR* dysfunction and/or as a consequence of a deterioration in the function of the organs affected. The objective of this review is to analyze all the possible endocrinological complications that can occur in patients with CF by evaluating the most recent papers in the literature.

## 1. Introduction

Cystic fibrosis (CF; OMIM # 219700) is a genetic autosomal recessive disease, caused by a defect in the cystic fibrosis transmembrane conductance regulator (CFTR) protein, an anionic channel present in the apical membrane of various epithelial cells, where it regulates electrolytic exchanges [1,2]. The main actions of the CFTR channel are the secretion of water in the epithelial tissues by generating secretions of chloride and the maintenance of depolarization in the plasma membrane through the efflux of chloride [1]. The *CFTR* gene coding for this protein is found on the long arm of chromosome 7 (7q.31.2; OMIM * 602421) [3]. More than 2000 gene mutations are known to cause the disease; the *F508del* mutation is the most frequent [4] and accounts for about two-thirds of mutated alleles in northern European and North American cohorts [5]. In Italy, it is less frequent; it is present in fewer than 50% of patients with CF [6].

CF is a multisystemic and progressive disease, making it fundamental for patients to receive an early diagnosis so that therapeutic and prophylactic measures can be implemented. Patients diagnosed during neonatal screening have better clinical outcomes and experience a slower progression of the disease [7,8,9]. The most affected apparatuses are the respiratory system, which presents recurrent infections, and the gastrointestinal system, with clinical manifestations that include recurrent meconium ileus at birth and malabsorption, which leads to growth retardation caused by progressive pancreatic insufficiency [10]. The life expectancy of individuals with CF remains variable, even though. In recent decades, new pharmacological therapies have increased the lifespans of many patients [11]. The main clinical manifestations of a deficit in *CFTR* protein are connected with altered exocrine glandular function in the pancreas and the dysfunction of pulmonary defense mechanisms, but other systems may be involved [10]. Patients can present with endocrinological conditions, such as diabetes, thyroid disorders, poor weight gain and linear growth, pubertal delay and hypogonadism, as well as with bone pathologies [12]. It is now widely recognized that more severe endocrinological complications are associated with a greater loss of functionality in the respiratory and gastrointestinal systems [13]. For example, worsening clinical symptoms of pulmonary distress and a decreased forced expiratory volume in the first second (FEV_1_) have been associated with the onset of diabetes [14]. 

The aim of this review is to analyze the endocrinological complications associated with CF and their possible impact on the clinical status of patients.

## 2. Cystic Fibrosis-Related Diabetes (CFRD)

### 2.1. Epidemiology

The prevalence of CFRD increases with age and is present in 2% of children, 19% of adolescents and 40–50% of adults with CF [15]. According to the European Foundation for Cystic Fibrosis Patient Registry, the prevalence of CFRD in 2015 was 0.8% in patients under the age of 10; 9.7% between 10 and 19 years; 24.1% between 20 and 29 years; and 32.7% in individuals aged ≥30 years [16].

Yi Y et al. [17] found that 39% of children between 3 months and 5 years with CF have reduced glucose tolerance and many children with normal tolerance have reduced stimulated insulin secretion after 2 years of age [18].

Among the predisposing factors for the development of diabetes are severe *CFTR* mutations (80% of subjects with severe mutations develop CFRD after the age of 40 [19]), a family history of type 2 diabetes, systemic use of corticosteroids, organ transplantation, CF-related liver disease and the development of pancreatic insufficiency [20].

A slowdown in growth may indicate diabetes. The appearance of CFRD can be preceded and followed by reduced gains in height and weight [14].

The prevalence of CFRD may well be set to fall following the introduction of new drugs that modulate the CFTR protein and prevent complications associated with compromised glucose metabolism [15].

### 2.2. Pathophysiology

There are many hypotheses regarding the mechanism by which diabetes occurs in patients with CF. 

CFRD has characteristics of both type 1 and type 2 diabetes mellitus, as it is caused by both a quantitative deficiency of insulin and the development of peripheral resistance to the hormone’s action [15].

The typical characteristics of CFRD are partial loss of insulin production and secretion due to loss/dysfunction of pancreatic beta cells, fluctuating levels of insulin resistance secondary to the state of chronic inflammation and an absence of an autoimmune status shown by levels of autoantibody concentration that are comparable to the general population [21].

The loss of pancreatic cells is secondary to pancreatic fibrotic progression. The abnormal functioning of the anion channels causes the formation and consequent accumulation of viscous material, which leads to damage to and obstruction of the exocrine pancreatic ducts, with subsequent fibrosis and deposition of amyloid in the pancreatic islets. This process causes the loss of pancreatic alpha and beta cells and a consequent reduction in insulin secretion [21].

In some studies [22,23,24], the overall mass of pancreatic islets was found to be uniformly reduced in the autopsy findings of patients with CF with and without CFRD. This would suggest that the development of diabetes is not directly caused by islet loss, but to intrinsic mechanisms of the pancreatic cells of patients with CF. It has been hypothesized that the mutation in the *CFTR* channel has a negative impact on insulin secretion as it could affect the activation process of intracellular insulin granules and calcium flow by influencing the activity of the Anoctamin-1 protein (ANO1), a voltage-sensitive calcium-activated chlorine channel, which participates in the depolarization of membranes and, thus, in the normal secretion of insulin [25].

One study found that mice with a *CFTR* mutation were prone to developing diabetes after mild beta-cell injury [26].

Genetic factors also appear to be implicated in the development of CFRD. Susceptibility genes for type 2 diabetes have been related to CFRD, specifically *CAPN10* (calpain 10; OMIM * 605286) and *TCF7L2* (transcription factor 7-like 2; OMIM * 602228) [27].

### 2.3. Diagnosis

The guidelines for diagnosis were drawn up jointly by the US Cystic Fibrosis Foundation (CFF), the American Diabetes Association (ADA) and the Pediatric Endocrine Society (PES) [28].

The diagnosis of CFRD is based on the same criteria as other forms of diabetes, specifically: random blood glucose ≥ 200 mg/dl (or ≥11.1 mmol/L) in the presence of suggestive symptoms, such as polyuria/polydipsia, blood glucose level ≥ 200 mg/dL (or ≥11.1 mmol/L) 2 h after an oral-glucose-tolerance test (OGTT), fasting blood glucose ≥ 126 mg/dl (or ≥7 mmol/L) or the finding of glycated hemoglobin ≥ 6.5% (or ≥48 mmol/mol). Any abnormal results must be confirmed by repeating the test on a separate day [28]. 

The CFF also recommends annual screening using OGTT from the age of 10 at a dose of 1.75 g/kg with a maximum of 75 g, after an overnight fast of at least 8 h [28].

### 2.4. Therapy

The glycemic goals in patients with CFRD are the same as those for patients with other types of diabetes [28]. 

The first step in controlling diabetes is commonly diet therapy. There are, however, specific guidelines for patients with CF [28]. Carbohydrate restriction is not recommended, and the elevated fat, salt, and calorie diet recommended for CF should be continued to avoid malnutrition.

The only pharmacological therapy currently recommended for the treatment of CFRD is insulin [11]. In patients with CFRD without fasting hyperglycemia it is possible to use an insulin scheme that includes only meal covers; if fasting hyperglycemia is present, the bolus scheme with the integration of basal insulin in the evening should be implemented [29].

Recent observations suggest that basal insulin alone may be efficacious in patients with CFRD without fasting hyperglycemia [30]. 

Insulin can also be administered by a continuous pump, which has been demonstrated to be more effective in covering glycemic fluctuations due to snacking during the day [31].

The insulin requirement in patients with CFRD is 0.5–0.8 IU/kg/day, with a greater need for coverage in boluses than in basal administrations. [32] As insulin sensitivity decreases over the years, current recommendations indicate starting insulin therapy at a low dose and increasing the dosage over time if necessary [33].

As malnutrition is a significant risk for patients with CF, it is advisable to modulate insulin dosage to suit carbohydrate intake rather than planning a rigid diet with carbohydrate restriction.

In the course of acute intercurrent illness, patients with CF become extremely insulin-resistant. In such cases, as well as during corticosteroid therapy, it may be necessary to increase the overall insulin dose by 30–40% [34].

Insulin sensitizers are not recommended because of their gastrointestinal side effects (metformin) and the increased risk of osteoporosis (thiazoidinediones) [28].

Other therapeutic options have been suggested for patients with CFRD, including glucagon-like peptide 1 (GLP-1) analogs, which are effective for treating patients with type 2 diabetes [35]. According to Geyer MC et al. [36], the use of GLP-1 analogues can result in reduced glycemic oscillation secondary to delays in gastric emptying. Concerns have arisen about the potential risk of pancreatitis associated with this drug. A possible solution could be the use of dipeptidyl peptidase 4 (DPP-4) inhibitors, which increase endogenous GLP-1 and do not have the same potentially serious side effects, but currently there are no studies in the literature to support their use.

The use of acarbose is not recommended as its action consists in reducing the absorption of carbohydrates, essential i for patients with CF to maintain a correct nutritional state.

CFTR modulator drugs, such as lumacaftor and ivacaftor, have improved insulin secretion and glycemic control in patients with CF [37,38]. However, some patients have experienced hypoglycemic episodes following the introduction of these drugs, and regular blood glucose checks should be performed on patients to whom they are administered [38]. Despite their possible beneficial effects on glycemic balance, currently, they cannot be considered effective in the control of CFRD on their own.

### 2.5. Complications

CFRD is associated with increased morbidity and mortality. The most important complication is a worsening of CF symptoms, including the deterioration of lung function and the worsening of nutritional status. In patients with CFRD, the annual loss in BMI is estimated to be 0.31 kg/m^2^, which in turn is associated with increased pulmonary difficulties [39].

Compared to other types of diabetes, microvascular complications are less frequent and less severe in patients with CFRD, probably because of the minimal amount of endogenous insulin secretion in these patients. It is recommended that annual screening for microvascular complications begin 5 years after CFRD diagnosis or immediately if the time of onset is unknown [28].

Patients with CF who develop diabetes are twice as likely to experience pulmonary exacerbations as those who do not have this complication [40]. The increase in blood glucose concentrations leads to the acidification of the fluids of the respiratory-tract epithelia. This causes an increase in lactate production, which favors colonization by *Pseudomonas aeruginosa* [41].

Insulin resistance has also been correlated with worse quality of sleep. A recent study found that reduced insulin sensitivity leads to shorter total sleep time, longer sleep-onset latency, more frequent awakening after sleep onset and poorer sleep efficiency [42].

## 3. Cystic-Fibrosis-Related Bone Disease

### 3.1. Epidemiology & Pathogenesis

Patients with CF frequently present with bone involvement. The microarchitecture of cortical and trabecular bone is compromised in most patients with CF [43]. In a recent systematic review of adults with CF undergoing dual-energy X-ray absorptiometry (DEXA), reduced bone-mineral concentrations (BMD) (T-score < −1 and >−2.5) were observed in 38% of patients and osteoporosis (T-score < −2.5) in 23.5% [44]. Clearly, the T-score (defined as the standard deviation (SD) score of the observed BMD compared with that of a normal young adult) should not be used for children, adolescents and young adults in whom peak bone mass has not yet been reached [45]; however, in paediatrics, a Z-score is used to compare a child’s BMD with an age- and gender-matched normal population, often with the need for appropriate adjustments for height, bone age or pubertal status. In children, low bone density is defined as a BMD Z-score of less than or equal to −2.0 SD [45]. In patients younger than 20 years, DEXA should be measured at the lumbar spine, between L1 and L4.

The pathogenesis of bone disease in patients with CF is multifactorial. The *CFTR* gene mutation could play a direct role in reducing bone-mineral mass. In mice with two inactivated copies of the gene, alterations in bone microarchitecture, characterized by a reduction in trabecular thickness and cortical width compared to heterozygous and healthy mice, have been observed [46].

Improvements in the strength and quality of bone were noted in a study on rodent models following the introduction of drugs modulating the function of *CFTR,* although these findings are yet to be confirmed in humans [47].

Malabsorption and malnutrition play a major role in the deterioration of bone health. It is known that the *CFTR* mutation causes pancreatic exocrine insufficiency, resulting in the reduced absorption of fat-soluble vitamins, especially K and D, which are essential for bone mineral health [48]. Vitamin K is involved in osteocalcin carboxylation and bone formation, while vitamin D is essential for the intestinal absorption of calcium. Vitamin D deficiency leads to a reduced absorption of calcium, with consequent secondary hyperparathyroidism, which in turn causes increased bone resorption through the activation of osteoclasts [48].

Malnutrition prevents patients with CF from reaching peak bone mass, resulting in an early reduction in bone mineral density in adulthood [49]. 

As in the general population, low levels of physical activity promote a reduction in bone mass in patients with CF.

Glucocorticoids, often given to patients with CF to treat pulmonary conditions, as well as after organ transplantation, are also known to affect bone density. The risk of fracture in patients on steroid therapy increases earlier than the reduction in bone mass becomes visible by DEXA, indicating the presence of microarchitectural alterations that are difficult to observe using traditional diagnostic tools [50]. Glucocorticoids also reduce intestinal calcium absorption and lead to a reduction in kidney function. Low bone calcium levels lead to secondary hyperparathyroidism. In addition, steroids reduce the production of sex hormones and the secretion of growth hormone (GH), which further contributes to a reduction in total bone mass [51].

An association has been observed between recurrent lung infections and decreased bone mass. There seems to be a correlation between the number of osteoclasts and the concentration of tumor necrosis factor alpha (TNF-a) and between their activity and serum interleukin-6 (IL-6) [52].

Other possible risk factors for reductions in bone-mineral mass are depression, the use of proton pump inhibitors, organ transplantation and chronic liver disease.

Patients with CF show more marked bone-resorption activity than the general population and have higher-than-normal serum levels of C-terminal telopeptide (CTX), parathormone (PTH), 1-25-vitamin D and TNF-α. By contrast, their levels of bone formation markers, such as osteocalcin (OC), total alkaline phosphatase (T-ALP) and procollagen type I N-terminal propeptide (P1NP) are normal. These data suggest that patients with CF are unable to compensate for accelerated bone breakdown [53].

Sphingosine 1-phosphate (S1P) also plays a role in bone degeneration in patients with CF. S1P is a lysophospholipid that mediates the passage of osteoclast precursors from bone to blood via the S1PR1 (sphingosine-1-phosphate receptor 1) receptor [54]. A second receptor, S1PR2 (sphingosine-1-phosphate receptor 2), mediates the reverse passage of precursors to the bone. Active vitamin D plays a role in inhibiting the production of this second receptor, thus blocking bone resorption [55]. The dysfunction of the CFTR protein causes a reduction in S1P [56,57], which, combined with the reduced quantity of active vitamin D present in the sera of patients with CF, contributes to the increase in bone resorption.

Some studies found a reduction in osteoprotegerin (OPG) levels in patients with CF compared to healthy subjects [58]. Le Heron et al. [59] suggested that this could be due to an inflammation-mediated increase in bone resorption resulting from the loss of CFTR protein activity.

### 3.2. Screening

The suggested timing of the first DEXA screening varies according to the guidelines considered: the CF Foundation [59] recommends performing the first DEXA scan at 18 years, or at 8 years in the presence of risk factors such as a history of fractures, delayed puberty or FEV1 < 50%; the European and French guidelines recommend a first DEXA screening at 8–10 years [48,60].

In patients with overt osteoporosis on drug therapy, an annual DEXA is essential for monitoring therapy and for evaluating its duration. Bone-turnover markers can be used to detect non-adherence to therapy, although no guidelines recommend routine testing [61].

Although the association between bone disease and CF is known, it is good practice to rule out other causes of secondary osteoporosis through blood tests to measure blood count, transaminases, alkaline phosphatase, 25OH vitamin D, PTH, urinary calcium and phosphorus and sex hormones.

A reduction in bone-formation indices (serum bone-specific alkaline phosphatase, OC, P1NP) and an increase in bone-resorption indices (CTX and urine N-terminal telopeptide of type 1 collagen) has been observed in patients with CF, especially during infections, but currently there are no recommendations on their use in patients with CF [52].

### 3.3. Therapy and Management

The first recommended intervention is to carry out physical activity for at least 30–45 min at least three times a week, as recommended by European guidelines [48].

It is of fundamental importance to maintain an adequate dietary intake of calcium and other micronutrients, such as vitamin K, phosphorus and magnesium. 

There are various reasons for calcium deficiency in these patients: intrinsic alteration of intestinal permeability [62]; vitamin D deficiency; and calcium malabsorption not adequately corrected by pancreatic enzyme replacement therapy [63]. It is essential to check calcium levels at least once a year; European and French guidelines also suggest measuring calciuria annually with the aim of maintaining a urinary calcium/creatinine ratio of between 0.1 and 0.5. It may be useful to prescribe calcium-rich drinks as a supplement to allow a greater absorption of ions than with tablets [64].

Patients’ daily calcium intake should be adequate for age and, therefore, a calcium-rich diet is recommended. If a deficiency is revealed by blood tests, calcium can be integrated orally in order to maintain the daily intake within the ranges established by the European guidelines, specifically: between 0 and 6 months, 210 mg/day; between 7 and 12 months, 270 mg/day; between 1 and 3 years, 500 mg/day; between 4 and 8 years, 800 mg/day; between 9 and 18 years, 1300 mg/day. It is also important to avoid excessive intake of vitamin A, which could have a negative role in bone mineralization [65].

Serum 25OH vitamin D should be evaluated annually, preferably in winter [66]. The threshold values to be maintained vary according to the guidelines considered: according to the CF Foundation, values above 30 ng/mL should be maintained [59], while according to the European and French guidelines, values above 20 ng/mL are sufficient [48,60]. Vitamin D supplementation should be given together with enzyme-replacement therapy to achieve the desired hormone levels.

Both pediatric and adult patients should take 400–800 IU of vitamin D daily, while serum vitamin D 25OH should be measured annually and possibly 3 months after changing dosage [66].

If dietary control is not sufficient to guarantee good bone health, a pharmacological therapy should be introduced.

Vitamin K deficiency is due to malabsorption, liver disease and the frequent use of antibiotics [67]. Vitamin K is a carboxylase cofactor that transforms the non-carboxylated form of OC into its gamma-carboxylated form. A deficiency in the latter form is frequently found in patients with CF and reduced BMD [68,69,70].

Although the only published study on vitamin K supplementation at a single dose of 10 mg weekly did not show any improvement in BMD or in carboxylated OC levels, the daily administration of vitamin K1 (phylloquinone) is still recommended given its low organic storage capacity [71].

Pediatric patients who can benefit from drug therapy include those with a BMD DEXA Z-score of less than 2 in the spine or whole body minus the head and a history of post-traumatic or vertebral crush fracture. Patients with the same DEXA score who are waiting for or who have received a lung transplant or who are undergoing chronic glucocorticoid therapy at supraphysiological doses for more than three months can also benefit [72].

Bisphosphonates act by inhibiting bone resorption and, thus, inducing apoptosis of the osteoclasts. This leads to increased bone mass, but it appears that there is no reduction in the number of fractures. A Cochrane review of nine randomized controlled trials in individuals with CF concluded that bisphosphonates taken orally and intravenously added mineral bone mass; however, the data did not reveal a significant reduction in the number of fractures these patients experienced [73].

Bisphosphonates can be administered both orally and intravenously, although the former is poorly absorbed. Alendronate, available as a liquid formulation, is the only orally administered bisphosphonate to have been studied in children with CF. Pamidronate is currently the intravenous formulation most commonly used in children, but as zolendronic acid is administered annually, it is slowly replacing pamidronate as the drug of choice [72].

For patients taking bisphosphonates, annual checks on bone-mineral mass with DEXA are recommended.

Denosumab is a monoclonal antibody against the receptor activator of nuclear factor kb ligand (RANKL) that works by blocking the activation of osteoclasts. Unlike bisphosphonates, it can be used in patients with chronic renal failure and does not require a preliminary measurement of creatinine. There are currently no published studies on the use of this drug in patients with CF, but as CFTR may play a role in mediating osteoprotegerin and RANKL in bone, its use could be considered. Denosumab could act on the intrinsic abnormalities of bone turnover resulting from the dysfunction of the CFTR protein.

Currently, however, Denosumab must be considered a second-line therapy in light of concerns about its side effects, such as atypical fractures of the femur, osteonecrosis of the jaw and the suppression of bone turnover after prolonged use.

SERMs (raloxifene in the USA and bazedoxifene in Europe and Japan) are antiresorptive drugs used in postmenopausal women and have not been studied in the CF population. These should never be used in the pediatric population [72].

A single study showed a 7–11% increase in BMD after treatment with teriparatide. As concerns emerged about the possibility that the drug increases the risk of osteosarcoma, its use has been banned in pediatric patients whose growth plates are still open and in patients with bone metastases, Paget’s disease and a history of radiation therapy [72].

## 4. Growth Failure

### 4.1. Epidemiology and Pathogenesis

Poor growth and developmental problems in children and adolescents with CF are common. Short stature is defined as a height Z-score of less than −2 standard deviations according to the centile tables of the population considered. 

Data from a review dating to 1993 reveal that short stature affects about 20% of subjects with CF [74]. More recent US data indicate that 11.9% of patients with CF are below the 10th percentile and 9.8% below the 5th [75]. However, recent data suggest an improvement in growth over the last few years [76].

Despite improved care over the past few decades and newborn screening, growth throughout childhood is still impaired in many patients with CF [77]. Subsequently, these patients maintain a regular growth trend until puberty, although they remain in the lower percentiles [78]. During adolescence, there is usually a severe restriction of growth with delayed puberty and spurts, delays in bone maturation and a reduction in definitive height [79].

The intrinsic mechanisms of the underlying disease seem to determine the height outcomes for patients with CF. A reduced mean neonatal length (with and without adjustment for gestational age) and reduced blood levels of insulin-like growth factor 1 (IGF-1) have been found in patients with CF [80]. A peripheral resistance to GH was hypothesized in the light of low basal and post-stimulus plasma levels of IGF-1 and IGFBP-3 in patients with CF compared to controls [81]. 

Growth restriction in mouse models with a loss of function of the *CFTR* gene at the neuronal level suggests that the protein has a direct role in regulating GH secretion and action [82]. These observations suggest that the impact of CF on height gain starts in the prenatal period and is dependent on pathophysiological mechanisms that are not yet fully understood.

The timing of the diagnosis appears to influence the growth outcomes of children with CF. Recent data show that patients with a late diagnosis achieve lower weight, height, and BMI than those who receive a diagnosis at newborn screening [83].

No statistically significant differences were observed in IGF-I, *GHR* gene expression or GHBP levels during childhood in children diagnosed early and those diagnosed late [83].

**Figure 1:**
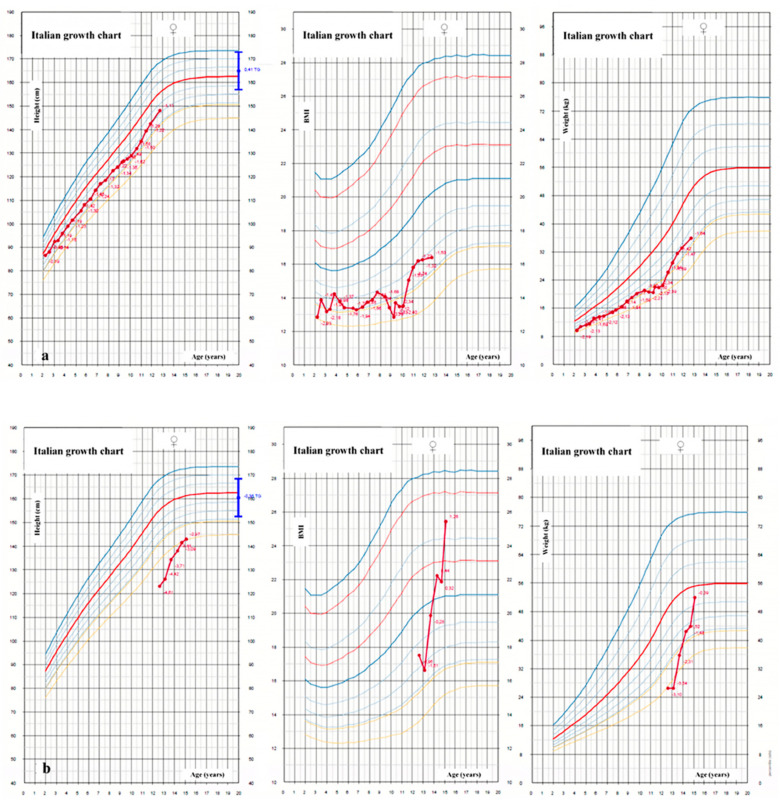
Height, weight and body-mass index (BMI) growth charts of two girls with cystic fibrosis between the ages of 2 and 18 years: a girl with pancreatic insufficiency with good compensation and normal puberty (**a**) and a girl with severe short stature and pubertal delay and pancreatic insufficiency undiagnosed until 12 years of age (**b**).

Malabsorption and malnutrition also affect growth (Figure 1a,b). Reduced caloric intake is frequent in CF patients and caused by anorexia secondary to the state of chronic inflammation, cough, and abdominal pain. Patients also have a high energy expenditure, a reduced absorption of nutrients in the intestine due to low levels of hydrochloric acid secretion and, in patients who have undergone intestinal resection for meconium ileus, a reduced intestinal absorbent surface with dysmotility. Pancreatic insufficiency also inevitably leads to a reduction in the absorption of fat-soluble vitamins, proteins and fats.

Patients with CF have reduced leptin secretion, which compromises the regulation of the GH at the hypothalamic level through the leptin receptor and through neuropeptide Y. Leptin suppresses the secretion of neuropeptide Y, which in turn inhibits the secretion of GH [84].

Chronic inflammation can affect growth through the alteration of the GH-IGF-1 axis. IL-6 interferes with the action of GH by disrupting its major signaling pathway (JAK-STAT) and IL-1 disrupts the expression of STAT5 and STAT3 [85].

The therapies used to control CF (including systemic and inhaler-administered glucocorticoids) can lead to growth failure via multiple pathways that interfere with GH secretion and its effect on the formation of collagen and bone and in nitrogen retention [86].

Chronic insulin deficiency may contribute to poor linear growth due to its anabolic effect [87,88].

### 4.2. Recommendations

Growth in pediatric patients with CF should be closely monitored (with follow-up visits every 6 months) to evaluate any deflections in growth rate or the passage to a lower percentile. The percentiles of height, weight and body-mass index (BMI) should be calculated using the WHO growth charts for children under 2 years [89] and using those of the CDC [90] (or those of Cacciari et al. in Italy [91]) for children between 2 and 20 years of age. 

Drugs commonly used to treat CF, such as glucocorticoids, should be administered for the shortest time necessary at the lowest effective dose.

It is always essential to investigate all the possible resolvable causes associated with CF that could influence growth, such as CFRD.

### 4.3. Therapy

Two recent meta-analyses [92,93] concluded that the use of recombinant GH (rhGH) can increase the height and lean body mass of patients with CF and short stature by 0.2–0.6 standard deviations. Drugs that modulate the *CFTR* gene seem to have a positive effect on height and weight. A recent study on children treated with ivafactor showed more significant height and weight gain compared to controls. In particular, the weight and height z-scores increased significantly from baseline to 26–48 weeks (1.08–2.10 cm/year height increase) [94]. Currently, it is not possible to draw similar conclusions about other modulator drugs due to a lack of studies on the subject and the more severe target genotype of these drugs.

## 5. Delayed Puberty

### 5.1. Diagnosis and Pathogenesis

Delayed puberty is defined in girls as the absence of breast maturation at age 13 or, more generally, by 2 to 2.5 standard deviations from the general population mean. The appearance of pubic hair is not considered indicative of puberty, since it depends on the maturation of the adrenal gland, which occurs separately from the activation of the hypothalamus–pituitary–gonadal axis. Any girl who has not had menses within 4 years of thelarche or who has primary amenorrhea at 16 years should undergo auxological testing [95].

In males, delayed puberty is defined as an increase in testicular volume below 4 mL after the age of 14 years or, by 2 to 2.5 standard deviations from the population mean. Males are often referred to a pediatric endocrinologist for poor growth between the ages of 11 and 13 years due to the absence of a pubertal spurt [95].

Delayed puberty may result from a failure of the hypothalamus–pituitary–gonadal (HPG) axis to mature (hypogonadotropic hypogonadism) or from primary gonadal dysfunction (hypergonadotropic hypogonadism). In the first case, there are often triggering causes such as low weight. Low levels of leptin have been associated with low levels of gonadotropins [96]. Menarche is also associated with reaching a certain weight [97]. 

The delayed puberty in patients with CF may be connected to malnutrition resulting from the underlying disease (Figure 1b) [95]. Other factors that contribute to late puberty in patients with CF are chronic inflammation and prolonged therapy based on corticosteroids [98].

Delays in testicular development or the onset of menarche [99] and of the pubertal spurt [100,101] has been documented in a number of studies. 

Landon et al. [102] showed that in patients with low serum levels of linoleic acid, oral supplementation and a dietary regime aimed at improving the nutritional state can result in weight gain, the resolution of amenorrhea and a normalization of menstrual irregularities in some patients.

CFTR knockout mice have been shown to have delayed puberty, as evidenced by a delayed vaginal opening age compared to healthy mice [103]. The role played by the CFTR protein in the regulation of GnRH secretion has been shown in in vitro studies [104], which demonstrate that the secretion of the hormone is blocked by the inhibition of CFTR in GNRH cell lines.

The literature on puberty in patients with CF is not conclusive. Recent observations indicate that CF patients reach the various Tanner’s stages and menarche at ages comparable to the general population [105,106]. A 2012 French work on a cohort of children with CF showed a timing of peak height velocity that was comparable to that of the control population [107].

Another recent study concluded that puberty in patients with CF with satisfactory nutritional status is delayed by about 2 years, with the most significant delay in patients homozygous for *deltaF508* or with pathological OGTT during adolescence [101].

An earlier study suggested that the HPG axis might cause a delayed increase in estradiol and FSH, which would not reach normal values until the age of 16 years [108].

Despite the lack of clarity on the timing of puberty in patients with CF, it is reasonable to monitor development in order to recognize signs of developmental delay that can affect final height and psychological wellbeing. Some surveys show that in 60% of adolescents with CF and pubertal delay, the absence of signs of pubertal development is experienced as a major problem and can even lead to suicide [109].

### 5.2. Treatment

The treatment of delayed puberty involves the elimination of possible triggering causes such as, in patients with CF in particular, malnutrition. Improving nutritional status is essential to allow the normal mechanisms responsible for growth spurts and sexual development to be activated. It is also important to exclude the coexistence of underlying causes, which, once resolved, could allow normal pubertal development.

If a permanent cause of hypogonadotropic hypogonadism is identified, it is essential to intervene pharmacologically by administering exogenous hormones to achieve an adult stature in line with the familiar target and an adequate bone mass.

In females, estrogens are administered transdermally and the dose adjusted approximately every 6 months for 34 years [110]. After two years of estrogen therapy or after the onset of the first menstrual cycle, progesterone is added. 

In males, hormone replacement therapy involves the administration of testosterone. This can be administered intramuscularly or via transdermal gel. The first method is the most studied and used in clinical practice. The dosage is changed every 6–12 months in order to emulate a normally functioning and developing HPG axis [95].

Alternative methods of treating pubertal delay are aimed at mirroring human physiology in this delicate phase of life. Specifically, GnRH, hCG, kisspeptin-10 and recombinant FSH are administered [111].

In patients with poor growth, the study of the growth-hormone axis can be considered. Some authors report that the use of the biosynthetic hormone in otherwise healthy children is associated with improvements in height, weight and lean body mass [112]. However, the data are inconsistent, and the use of recombinant GH should be evaluated on a case-by-case basis.

## 6. Male Hypogonadism and Infertility

**Figure 2:**
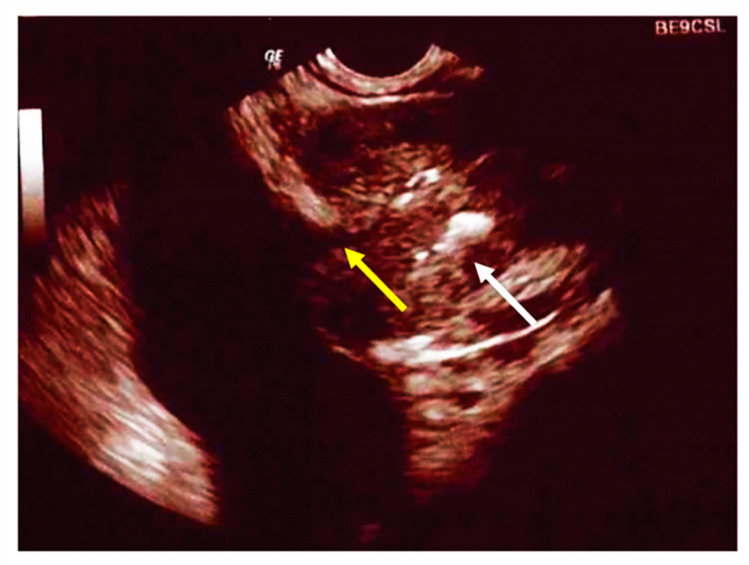
A transrectal ultrasound examination demonstrating calcification within the ejaculatory duct (**white arrow**) with dilatation of the vas deferens proximally (**yellow arrow**).

CF is associated with hypogonadism and male infertility. Studies have reported a 98% rate of infertility due to obstructive causes, specifically the congenital bilateral absence of the vas deferens (CBAVD) and the absence or atrophy of the seminal vesicles (Figure 2) [113]. However, patients with CF produce sperm and have histologically normal testes. The diagnosis of the condition is performed by transrectal ultrasound. 

Among the various causes related to infertility are a possible obstructive blockage of the seminal ducts due to the extreme viscosity of the semen [114] and the incorrect differentiation of Wolf’s ducts secondary to the *CFTR* mutation [115].

Hypogonadism is secondary to testosterone deficiency, which causes a delay in the acquisition of the secondary sexual characteristics typical of puberty and a reduction in muscle mass and bone-mineral density. Leifke et al. [116] suggest that approximately 25% of males with CF have low testosterone blood levels. 

There are currently no official recommendations on the clinical management of this condition. Hypogonadism associated with CF is multifactorial. Chronic inflammation and the overuse of corticosteroids can lead to both the dysregulation of the HPG axis and primary hypogonadism. Furthermore, CFTR is expressed both in hypothalamic cells and in vas deferens, epididymis, and Sertoli cells and, consequently, the gene mutation could be responsible for the pathological state [117].

The diagnosis involves measuring free and protein-bound testosterone. Blood collection should take place in the morning and not during acute intercurrent illness. The levels of LH and FSH can help to distinguish between primary and secondary hypogonadism, while the levels of sex hormone binding globulin (SHBG) can identify altered levels of testosterone due to causes that alter the plasma levels of this protein. In particular, low levels of SHBG are detectable in patients who are obese, with excess GH, with insulin resistance, with hypothyroidism and who use glucocorticoids. Conversely, high levels are observed in patients with hyperthyroidism, who use antiepileptic drugs or with liver disease [118].

The blood concentration of this hormone has a positive correlation with bone-mineral concentration and, consequently, lower values are associated with an increased number of X-ray-documented vertebral fractures. Therefore, including testosterone testing in follow-up for males with CF and osteoporosis and in patients with symptoms suggestive of hypogonadism is advisable. 

Testosterone can be replaced in various ways, the most common being transcutaneous gel and intramuscular injections. Dosage-control tests should be performed 2–3 months after starting therapy and then every 6–12 months. The therapeutic goal is to reach and maintain mid-normal blood levels, i.e., 400–700 ng/dL. Among the side effects associated with testosterone therapy are erythrocytosis, venous thromboembolism, cardiovascular events and sleep apnea [118].

## 7. Thyroid Function

Thyroid function in patients with CF has been studied over the past few decades, but the literature does not provide clear conclusions. The CFTR is present in the human thyroid epithelium [119].

One of the hypothesized mechanisms underlying the development of hypothyroidism in patients with CF is altered ion transport in the thyroid epithelium [120]. A reduced secretion of chloride activated by cAMP, an ion that can act as a counterion for the accumulation of iodine, leading to the interruption of the accumulation of iodine in the thyroid, has been observed within a completely preserved thyroid histology in a knockout porcine model [120].

Since the 1970s, a time when large quantities of iodine-based expectorants were administered to patients with CF, questions have been raised about the effects of iodine. According to Dolan et al. [121], 44% of patients undergoing this therapy developed goiters, and 83% of these developed hypothyroidism. Similar data were reported by Azizi et al. [122], who documented reductions in total plasma triiodothyronine concentrations (T_3_). These low levels of T_3_ could be explained in some cases by the state of malnutrition in patients with CF and, therefore, by selenium deficiency, which results in inadequate activity of hepatic deiodinase, with consequent insufficient synthesis and metabolism of the thyroid hormone [123]. A significant correlation was found between increased selenium levels and decreased TSH, while increases in T_3_ and decreases in the T_4_/T_3_ ratio have been reported in patients with CF treated with oral selenium [124].

Conflicting results are also present in the literature regarding the response of thyrotropin (TSH) to thyrotropin-releasing hormone (TRH) in patients with CF. Segall-Blanck et al. [125] reported a normal response, while De Luca et al. [126] described exaggerated responses.

A more recent study, by Volta et al. [127], performed on a small cohort of patients with CF did not reveal any thyroid dysfunction compared to controls. Among the hypotheses related to this finding, the authors described the improvement in the nutritional status of patients with CF since the 1970s and the discontinuation of the use of iodine-containing expectorants.

A study carried out following the interruption of the administration of iodine-based expectorants showed a reduction of 11% in pediatric subclinical hypothyroidism [128].

The most recent study in the literature on this topic dates back to 2015 and was carried out by Lee et al. [129], on a cohort of 89 patients. In total, 34% of the patients had abnormal T_4_ levels, even though the vast majority of the patients with altered levels did not present large alterations. A total of 27% of the subjects presented an abnormal response to the thyroid function test. The latter finding, however, could have been related to the chronic state of these patients’ underlying condition.

## 8. Conclusions

Endocrinological complications in patients with CF are frequent. A child diagnosed with CF must undergo a complete auxological evaluation every six months in order to detect anomalies in height and weight; according to the CFF guidelines, from the age of 10, an OGTT should also be performed annually to evaluate the possible occurrence of CFRD and a DEXA should be performed to assess bone mineralization. The timing of the repetition of the DEXA should depend on the detected Z-score (European CF Society).

The complications we discussed can require therapies that need careful follow-up, to evaluate not only clinical efficacy and the possible need for dosage adjustment, but also the possible appearance of unwanted effects.

The involvement of pediatricians experienced in endocrinology in the global management of patients with CF is of fundamental importance.

From our review of the literature, no unanimity of thought regarding thyroid-function anomalies has emerged so far, although current opinion leans towards the absence of a correlation with CF.

Further evaluations are essential to clarify the role of the mutation responsible for the underlying pathology in the pathogenesis of the complications we analyzed.

## Data Availability

Not applicable.

## References

[1] Sheppard D.N., Welsh M.J. (1999). Structure and function of the CFTR chloride channel. Physiol. Rev..

[2] Shteinberg M., Haq I.J., Polineni D., Davies J.C. (2021). Cystic fibrosis. Lancet.

[3] Rommens J.M., Iannuzzi M.C., Kerem B., Drumm M.L., Melmer G., Dean M., Rozmahel R., Cole J.L., Kennedy D., Hidaka N. (1989). Identification of the cystic fibrosis gene: Chromosome walking and jumping. Science.

[4] Elborn J.S. (2016). Cystic fibrosis. Lancet.

[5] Orenti A., Zolin A., Jung A., van Rens J., Fox A., Krasnyk M., Daneau G., Hatziagorou E., Mei-Zahav M., Naehrlich L. ECFSPR Annual Report 2019. European Cystic Fibrosis Society 2021. https://www.ecfs.eu/sites/default/files/general-content-files/working-groups/ecfs-patient-registry/ECFSPR_Report_2019_v1_16Feb2022.pdf.

[6] Terlizzi V., Claut L., Tosco A., Colombo C., Raia V., Fabrizzi B., Lucarelli M., Angeloni A., Cimino G., Castaldo A. (2021). A survey of the prevalence, management and outcome of infants with an inconclusive diagnosis following newborn bloodspot screening for cystic fibrosis (CRMS/CFSPID) in six Italian centres. J. Cyst. Fibros..

[7] Sims E.J., Clark A., McCormick J., Mehta G., Connett G., Mehta A., United Kingdom Cystic Fibrosis Database Steering Committee (2007). Cystic fibrosis diagnosed after 2 months of age leads to worse outcomes and requires more therapy. Pediatrics.

[8] Dijk F.N., McKay K., Barzi F., Gaskin K.J., Fitzgerald D.A. (2011). Improved survival in cystic fibrosis patients diagnosed by newborn screening compared to a historical cohort from the same centre. Arch. Dis. Child..

[9] Taccetti G., Botti M., Terlizzi V., Cavicchi M.C., Neri A.S., Galici V., Mergni G., Centrone C., Peroni D.G., Festini F. (2020). Clinical and Genotypical Features of False-Negative Patients in 26 Years of Cystic Fibrosis Neonatal Screening in Tuscany, Italy. Diagnostics.

[10] Orenstein D.M., Winnie G.B., Altman H. (2002). Cystic fibrosis: A 2002 update. J. Pediatr..

[11] Corriveau S., Sykes J., Stephenson A.L. (2018). Cystic fibrosis survival: The changing epidemiology. Curr. Opin. Pulm. Med..

[12] Ode K.L., Chan C.L., Granandos A., Putman M., Moheet A. (2019). Endocrine Complications of Cystic Fibrosis: A Multisystem Disease of the Endocrine Organs. Semin. Respir. Crit. Care Med..

[13] Moheet A., Moran A. (2017). CF-related diabetes: Containing the metabolic miscreant of cystic fibrosis. Pediatr. Pulmonol..

[14] Terliesner N., Vogel M., Steighardt A., Gausche R., Henn C., Hentschel J., Kapellen T., Klamt S., Gebhardt J., Kiess W. (2017). Cystic-fibrosis related-diabetes (CFRD) is preceded by and associated with growth failure and deteriorating lung function. J. Pediatr. Endocrinol. Metab..

[15] Moran A., Becker D., Casella S.J., Gottlieb P.A., Kirkman M.S., Marshall B.C., Slovis B., CFRD Consensus Conference Committee (2010). Epidemiology, pathophysiology, and prognostic implications of cystic fibrosis-related diabetes: A technical review. Diabetes Care.

[16] Olesen H.V., Drevinek P., Gulmans V.A., Hatziagorou E., Jung A., Mei-Zahav M., Stojnic N., Thomas M., Zolin A., ECFSPR Steering Group (2020). Cystic fibrosis related diabetes in Europe: Prevalence, risk factors and outcome. J. Cyst. Fibros..

[17] Yi Y., Norris A.W., Wang K., Sun X., Uc A., Moran A., Engelhardt J.F., Ode K.L. (2016). Abnormal Glucose Tolerance in Infants and Young Children with Cystic Fibrosis. Am. J. Respir. Crit. Care Med..

[18] Ode K.L., Frohnert B., Laguna T., Phillips J., Holme B., Regelmann W., Thomas W., Moran A. (2010). Oral glucose tolerance testing in children with cystic fibrosis. Pediatr. Diabetes.

[19] Lewis C., Blackman S.M., Nelson A., Oberdorfer E., Wells D., Dunitz J., Thomas W., Moran A. (2015). Diabetes-related mortality in adults with cystic fibrosis. Role of genotype and sex. Am. J. Respir. Crit. Care Med..

[20] Blackman S.M., Commander C.W., Watson C., Arcara K.M., Strug L.J., Stonebraker J.R., Wright F.A., Rommens J.M., Sun L., Pace R.G. (2013). Genetic modifiers of cystic fibrosis-related diabetes. Diabetes.

[21] Gottlieb P.A., Yu L., Babu S., Wenzlau J., Bellin M., Frohnert B.I., Moran A. (2012). No relation between cystic fibrosis-related diabetes and type 1 diabetes autoimmunity. Diabetes Care.

[22] Hart N.J., Aramandla R., Poffenberger G., Fayolle C., Thames A.H., Bautista A., Spigelman A.F., Babon J.A.B., DeNicola M.E., Dadi P.K. (2018). Cystic fibrosis-related diabetes is caused by islet loss and inflammation. JCI Insight.

[23] Soejima K., Landing B.H. (1986). Pancreatic islets in older patients with cystic fibrosis with and without diabetes mellitus: Morphometric and immunocytologic studies. Pediatr. Pathol..

[24] Bogdani M., Blackman S.M., Ridaura C., Bellocq J.P., Powers A.C., Aguilar-Bryan L. (2017). Structural abnormalities in islets from very young children with cystic fibrosis may contribute to cystic fibrosis-related diabetes. Sci. Rep..

[25] Edlund A., Esguerra J.L., Wendt A., Flodström-Tullberg M., Eliasson L. (2014). CFTR and Anoctamin 1 (ANO1) contribute to cAMP amplified exocytosis and insulin secretion in human and murine pancreatic beta-cells. BMC Med..

[26] Stalvey M.S., Muller C., Schatz D.A., Wasserfall C.H., Campbell-Thompson M.L., Theriaque D.W., Flotte T.R., Atkinson M.A. (2006). Cystic fibrosis transmembrane conductance regulator deficiency exacerbates islet cell dysfunction after beta-cell injury. Diabetes.

[27] Blackman S.M., Hsu S., Ritter S.E., Naughton K.M., Wright F.A., Drumm M.L., Knowles M.R., Cutting G.R. (2009). A susceptibility gene for type 2 diabetes confers substantial risk for diabetes complicating cystic fibrosis. Diabetologia.

[28] Moran A., Brunzell C., Cohen R.C., Katz M., Marshall B.C., Onady G., Robinson K.A., Sabadosa K.A., Stecenko A., Slovis B. (2010). Clinical care guidelines for cystic fibrosis-related diabetes: A position statement of the American Diabetes Association and a clinical practice guideline of the Cystic Fibrosis Foundation, endorsed by the Pediatric Endocrine Society. Diabetes Care.

[29] Moran A., Pekow P., Grover P., Zorn M., Slovis B., Pilewski J., Tullis E., Liou T.G., Allen H., Cystic Fibrosis Related Diabetes Therapy Study Group (2009). Insulin therapy to improve BMI in cystic fibrosis-related diabetes without fasting hyperglycemia: Results of the cystic fibrosis related diabetes therapy trial. Diabetes Care.

[30] Moran A., Pillay K., Becker D., Granados A., Hameed S., Acerini C.L. (2018). ISPAD Clinical Practice Consensus Guidelines 2018: Management of cystic fibrosis-related diabetes in children and adolescents. Pediatr. Diabetes.

[31] Hardin D.S., Rice J., Rice M., Rosenblatt R. (2009). Use of the insulin pump in treat cystic fibrosis related diabetes. J. Cyst. Fibros..

[32] Sunni M., Bellin M.D., Moran A. (2013). Exogenous insulin requirements do not differ between youth and adults with cystic fibrosis related diabetes. Pediatr. Diabetes.

[33] Colomba J., Boudreau V., Lehoux-Dubois C., Desjardins K., Coriati A., Tremblay F., Rabasa-Lhoret R. (2019). The main mechanism associated with progression of glucose intolerance in older patients with cystic fibrosis is insulin resistance and not reduced insulin secretion capacity. J. Cyst. Fibros..

[34] Rasouli N., Seggelke S., Gibbs J., Hawkins R.M., Casciano M.L., Cohlmia E., Taylor-Cousar J., Wang C., Pereira R., Hsia E. (2012). Cystic fibrosis-related diabetes in adults: Inpatient management of 121 patients during 410 admissions. J. Diabetes Sci. Technol..

[35] Kuo P., Stevens J.E., Russo A., Maddox A., Wishart J.M., Jones K.L., Greville H., Hetzel D., Chapman I., Horowitz M. (2011). Gastric emptying, incretin hormone secretion, and postprandial glycemia in cystic fibrosis—Effects of pancreatic enzyme supplementation. J. Clin. Endocrinol. Metab..

[36] Geyer M.C., Sullivan T., Tai A., Morton J.M., Edwards S., Martin A.J., Perano S.J., Gagliardi L., Rayner C.K., Horowitz M. (2019). Exenatide corrects postprandial hyperglycaemia in young people with cystic fibrosis and impaired glucose tolerance: A randomized crossover trial. Diabetes Obes. Metab..

[37] Kelly A., De Leon D.D., Sheikh S., Camburn D., Kubrak C., Peleckis A.J., Stefanovski D., Hadjiliadis D., Rickels M.R., Rubenstein R.C. (2019). Islet Hormone and Incretin Secretion in Cystic Fibrosis after Four Months of Ivacaftor Therapy. Am. J. Respir. Crit. Care Med..

[38] Bellin M.D., Laguna T., Leschyshyn J., Regelmann W., Dunitz J., Billings J., Moran A. (2013). Insulin secretion improves in cystic fibrosis following ivacaftor correction of CFTR: A small pilot study. Pediatr. Diabetes.

[39] Mauch R.M., Kmit A.H., Marson F.A., Levy C.E., Barros-Filho A.A., Ribeiro J.D. (2016). Association of growth and nutritional parameters with pulmonary function in cystic fibrosis: A literature review. Rev. Paul. Pediatr..

[40] Van Sambeek L., Cowley E.S., Newman D.K., Kato R. (2015). Sputum glucose and glycemic control in cystic fibrosis-related diabetes: A cross-sectional study. PLoS ONE.

[41] Garnett J.P., Kalsi K.K., Sobotta M., Bearham J., Carr G., Powell J., Brodlie M., Ward C., Tarran R., Baines D.L. (2016). Hyperglycaemia and Pseudomonas aeruginosa acidify cystic fibrosis airway surface liquid by elevating epithelial monocarboxylate transporter 2 dependent lactate-H^+^ secretion. Sci. Rep..

[42] Simon S.L., Vigers T., Campbell K., Pyle L., Branscomb R., Nadeau K.J., Chan C.L. (2018). Reduced insulin sensitivity is correlated with impaired sleep in adolescents with cystic fibrosis. Pediatr. Diabetes.

[43] Putman M.S., Milliren C.E., Derrico N., Uluer A., Sicilian L., Lapey A., Sawicki G., Gordon C.M., Bouxsein M.L., Finkelstein J.S. (2014). Compromised bone microarchitecture and estimated bone strength in young adults with cystic fibrosis. J. Clin. Endocrinol. Metab..

[44] Paccou J., Zeboulon N., Combescure C., Gossec L., Cortet B. (2010). The prevalence of osteoporosis, osteopenia, and fractures among adults with cystic fibrosis: A systematic literature review with meta-analysis. Calcif. Tissue Int..

[45] Laine C.M., Laine T. (2013). Diagnosis of Osteoporosis in Children and Adolescents. Eur. Endocrinol..

[46] Dif F., Marty C., Baudoin C., de Vernejoul M.C., Levi G. (2004). Severe osteopenia in CFTR-null mice. Bone.

[47] Jacquot J., Delion M., Gangloff S., Braux J., Velard F. (2016). Bone disease in cystic fibrosis: New pathogenic insights opening novel therapies. Osteoporos. Int..

[48] Sermet-Gaudelus I., Bianchi M.L., Garabédian M., Aris R.M., Morton A., Hardin D.S., Elkin S.L., Compston J.E., Conway S.P., Castanet M. (2011). European cystic fibrosis bone mineralisation guidelines. J. Cyst. Fibros..

[49] Sharma S., Jaksic M., Fenwick S., Byrnes C., Cundy T. (2017). Accrual of Bone Mass in Children and Adolescents with Cystic Fibrosis. J. Clin. Endocrinol. Metab..

[50] Adami G., Saag K.G. (2019). Glucocorticoid-induced osteoporosis: 2019 concise clinical review. Osteoporos. Int..

[51] Mazziotti G., Giustina A. (2013). Glucocorticoids and the regulation of growth hormone secretion. Nat. Rev. Endocrinol..

[52] Shead E.F., Haworth C.S., Barker H., Bilton D., Compston J.E. (2010). Osteoclast function, bone turnover and inflammatory cytokines during infective exacerbations of cystic fibrosis. J. Cyst. Fibros..

[53] Mora Vallellano J., Delgado Pecellín C., Delgado Pecellín I., Quintana Gallego E., López-Campos J.L. (2021). Evaluation of bone metabolism in children with cystic fibrosis. Bone.

[54] Ishii M., Kikuta J., Shimazu Y., Meier-Schellersheim M., Germain R.N. (2010). Chemorepulsion by blood S1P regulates osteoclast precursor mobilization and bone remodeling in vivo. J. Exp. Med..

[55] Kikuta J., Kawamura S., Okiji F., Shirazaki M., Sakai S., Saito H., Ishii M. (2013). Sphingosine-1-phosphate-mediated osteoclast precursor monocyte migration is a critical point of control in antibone-resorptive action of active vitamin D. Proc. Natl. Acad. Sci. USA.

[56] Worgall T.S., Veerappan A., Sung B., Kim B.I., Weiner E., Bholah R., Silver R.B., Jiang X.C., Worgall S. (2013). Impaired sphingolipid synthesis in the respiratory tract induces airway hyperreactivity. Sci. Transl. Med..

[57] Xu Y., Krause A., Limberis M., Worgall T.S., Worgall S. (2013). Low sphingosine-1-phosphate impairs lung dendritic cells in cystic fibrosis. Am. J. Respir. Cell. Mol. Biol..

[58] Le Heron L., Guillaume C., Velard F., Braux J., Touqui L., Moriceau S., Sermet-Gaudelus I., Laurent-Maquin D., Jacquot J. (2010). Cystic fibrosis transmembrane conductance regulator (CFTR) regulates the production of osteoprotegerin (OPG) and prostaglandin (PG) E2 in human bone. J. Cyst. Fibros..

[59] Aris R.M., Merkel P.A., Bachrach L.K., Borowitz D.S., Boyle M.P., Elkin S.L., Guise T.A., Hardin D.S., Haworth C.S., Holick M.F. (2005). Guide to bone health and disease in cystic fibrosis. J. Clin. Endocrinol. Metab..

[60] Sermet-Gaudelus I., Castanet M., Souberbielle J.C., Mallet E., Le Groupe de Travail sur Minéralisation Osseuse et Mucoviscidose de la Fédération Française des Centres de Ressource et de Compétence en Mucoviscidose (2009). Minéralisation osseuse et mucoviscidose [Bone health in cystic fibrosis]. Arch. Pediatr..

[61] Eastell R., Rosen C.J., Black D.M., Cheung A.M., Murad M.H., Shoback D. (2019). Pharmacological Management of Osteoporosis in Postmenopausal Women: An Endocrine Society* Clinical Practice Guideline. J. Clin. Endocrinol. Metab..

[62] Schulze K.J., O’brien K.O., Germain-Lee E.L., Baer D.J., Leonard A.L., Rosenstein B.J. (2003). Endogenous fecal losses of calcium compromise calcium balance in pancreatic-insufficient girls with cystic fibrosis. J. Pediatr..

[63] Aris R.M., Lester G.E., Dingman S., Ontjes D.A. (1999). Altered calcium homeostasis in adults with cystic fibrosis. Osteoporos. Int..

[64] Cheng S., Lyytikäinen A., Kröger H., Lamberg-Allardt C., Alén M., Koistinen A., Wang Q.J., Suuriniemi M., Suominen H., Mahonen A. (2005). Effects of calcium, dairy product, and vitamin D supplementation on bone mass accrual and body composition in 10-12-y-old girls: A 2-y randomized trial. Am. J. Clin. Nutr..

[65] Maurel D.B., Boisseau N., Benhamou C.L., Jaffre C. (2012). Alcohol and bone: Review of dose effects and mechanisms. Osteoporos. Int..

[66] Tangpricha V., Kelly A., Stephenson A., Maguiness K., Enders J., Robinson K.A., Marshall B.C., Borowitz D., Cystic Fibrosis Foundation Vitamin D Evidence-Based Review Committee (2012). An update on the screening, diagnosis, management, and treatment of vitamin D deficiency in individuals with cystic fibrosis: Evidence-based recommendations from the Cystic Fibrosis Foundation. J. Clin. Endocrinol. Metab..

[67] Rashid M., Durie P., Andrew M., Kalnins D., Shin J., Corey M., Tullis E., Pencharz P.B. (1999). Prevalence of vitamin K deficiency in cystic fibrosis. Am. J. Clin. Nutr..

[68] Conway S.P., Wolfe S.P., Brownlee K.G., White H., Oldroyd B., Truscott J.G., Harvey J.M., Shearer M.J. (2005). Vitamin K status among children with cystic fibrosis and its relationship to bone mineral density and bone turnover. Pediatrics.

[69] Aris R.M., Ontjes D.A., Brown S.A., Chalermskulrat W., Neuringer I., Lester G.E. (2003). Carboxylated osteocalcin levels in cystic fibrosis. Am. J. Respir. Crit. Care Med..

[70] Fewtrell M.S., Benden C., Williams J.E., Chomtho S., Ginty F., Nigdikar S.V., Jaffe A. (2008). Undercarboxylated osteocalcin and bone mass in 8-12 year old children with cystic fibrosis. J. Cyst. Fibros..

[71] Jagannath V.A., Fedorowicz Z., Thaker V., Chang A.B. (2015). Vitamin K supplementation for cystic fibrosis. Cochrane Database Syst. Rev..

[72] Putman M.S., Anabtawi A., Le T., Tangpricha V., Sermet-Gaudelus I. (2019). Cystic fibrosis bone disease treatment: Current knowledge and future directions. J. Cyst. Fibros..

[73] Conwell L.S., Chang A.B. (2012). Bisphosphonates for osteoporosis in people with cystic fibrosis. Cochrane Database Syst. Rev..

[74] Lai H.C., Kosorok M.R., Sondel S.A., Chen S.T., FitzSimmons S.C., Green C.G., Shen G., Walker S., Farrell P.M. (1998). Growth status in children with cystic fibrosis based on the National Cystic Fibrosis Patient Registry data: Evaluation of various criteria used to identify malnutrition. J. Pediatr..

[75] Cystic Fibrosis Foundation (2016). Cystic Fibrosis Foundation Patient Registry. Annual Data Report.

[76] Cystic Fibrosis Foundation (2017). Cystic Fibrosis Foundation Patient Registry. Annual Data Report.

[77] Leung D.H., Heltshe S.L., Borowitz D., Gelfond D., Kloster M., Heubi J.E., Stalvey M., Ramsey B.W., Baby Observational and Nutrition Study (BONUS) Investigators of the Cystic Fibrosis Foundation Therapeutics Development Network (2017). Effects of Diagnosis by Newborn Screening for Cystic Fibrosis on Weight and Length in the First Year of Life. JAMA Pediatr..

[78] Karlberg J., Kjellmer I., Kristiansson B. (1991). Linear growth in children with cystic fibrosis. I. Birth to 8 years of age. Acta Paediatr. Scand..

[79] Blackman S.M., Tangpricha V. (2016). Endocrine Disorders in Cystic Fibrosis. Pediatr. Clin. N. Am..

[80] Haeusler G., Frisch H., Waldhör T., Götz M. (1994). Perspectives of longitudinal growth in cystic fibrosis from birth to adult age. Eur. J. Pediatr..

[81] Laursen E.M., Lanng S., Rasmussen M.H., Koch C., Skakkebaek N.E., Müller J. (1999). Normal spontaneous and stimulated GH levels despite decreased IGF-I concentrations in cystic fibrosis patients. Eur. J. Endocrinol..

[82] Hodges C.A., Grady B.R., Palmert M.R., Drumm M.L. (2010). Loss of cftr function in neurons results in poor growth and possible endocrine dysfunction. Pediatr. Pulmonol..

[83] Pagani S., Bozzola E., Acquafredda G., Terlizzi V., Raia V., Majo F., Villani A., Bozzola M. (2019). GH-IGF-1 Axis in Children with Cystic Fibrosis. Clin. Med. Res..

[84] Hawkes C.P., Grimberg A. (2015). Insulin-Like Growth Factor-I is a Marker for the Nutritional State. Pediatr. Endocrinol. Rev..

[85] Cirillo F., Lazzeroni P., Sartori C., Street M.E. (2017). Inflammatory Diseases and Growth: Effects on the GH-IGF Axis and on Growth Plate. Int. J. Mol. Sci..

[86] Allen D.B. (1996). Growth suppression by glucocorticoid therapy. Endocrinol. Metab. Clin. N. Am..

[87] Ripa P., Robertson I., Cowley D., Harris M., Masters I.B., Cotterill A.M. (2002). The relationship between insulin secretion, the insulin-like growth factor axis and growth in children with cystic fibrosis. Clin. Endocrinol..

[88] Cheung M.S., Bridges N.A., Prasad S.A., Francis J., Carr S.B., Suri R., Balfour-Lynn I.M. (2009). Growth in children with cystic fibrosis-related diabetes. Pediatr. Pulmonol..

[89] WHO Multicentre Growth Reference Study Group (2006). WHO Child Growth Standards based on length/height, weight and age. Acta Paediatr. Suppl..

[90] Ziegler E.E. (2015). 4.2 The CDC and Euro Growth Charts. World Rev. Nutr. Diet..

[91] Cacciari E., Milani S., Balsamo A., Spada E., Bona G., Cavallo L., Cerutti F., Gargantini L., Greggio N., Tonini G. (2006). Italian cross-sectional growth charts for height, weight and BMI (2 to 20 yr). J. Endocrinol. Investig..

[92] Thaker V., Haagensen A.L., Carter B., Fedorowicz Z., Houston B.W. (2015). Recombinant growth hormone therapy for cystic fibrosis in children and young adults. Cochrane Database Syst. Rev..

[93] Phung O.J., Coleman C.I., Baker E.L., Scholle J.M., Girotto J.E., Makanji S.S., Chen W.T., Talati R., Kluger J., White C.M. (2010). Recombinant human growth hormone in the treatment of patients with cystic fibrosis. Pediatrics.

[94] Stalvey M.S., Pace J., Niknian M., Higgins M.N., Tarn V., Davis J., Heltshe S.L., Rowe S.M. (2017). Growth in Prepubertal Children With Cystic Fibrosis Treated With Ivacaftor. Pediatrics.

[95] Goldsweig B., Kaminski B., Sidhaye A., Blackman S.M., Kelly A. (2019). Puberty in cystic fibrosis. J. Cyst. Fibros..

[96] Welt C.K., Chan J.L., Bullen J., Murphy R., Smith P., DePaoli A.M., Karalis A., Mantzoros C.S. (2004). Recombinant human leptin in women with hypothalamic amenorrhea. N. Engl. J. Med..

[97] Frisch R.E., Revelle R. (1971). Height and weight at menarche and a hypothesis of menarche. Arch. Dis. Child..

[98] Wong S.C., Dobie R., Altowati M.A., Werther G.A., Farquharson C., Ahmed S.F. (2016). Growth and the Growth Hormone-Insulin Like Growth Factor 1 Axis in Children With Chronic Inflammation: Current Evidence, Gaps in Knowledge, and Future Directions. Endocr. Rev..

[99] Mitchell-Heggs P., Mearns M., Batten J.C. (1976). Cystic fibrosis in adolescents and adults. Q. J. Med..

[100] Landon C., Rosenfeld R.G. (1984). Short stature and pubertal delay in male adolescents with cystic fibrosis. Androgen treatment. Am. J. Dis. Child..

[101] Johannesson M., Gottlieb C., Hjelte L. (1997). Delayed puberty in girls with cystic fibrosis despite good clinical status. Pediatrics.

[102] Landon C., Kerner J.A., Castillo R., Adams L., Whalen R., Lewiston N.J. (1981). Oral correction of essential fatty acid deficiency in cystic fibrosis. JPEN J. Parenter. Enteral. Nutr..

[103] Jin R., Hodges C.A., Drumm M.L., Palmert M.R. (2006). The cystic fibrosis transmembrane conductance regulator (Cftr) modulates the timing of puberty in mice. J. Med. Genet..

[104] Weyler R.T., Yurko-Mauro K.A., Rubenstein R., Kollen W.J., Reenstra W., Altschuler S.M., Egan M., Mulberg A.E. (1999). CFTR is functionally active in GnRH-expressing GT1-7 hypothalamic neurons. Am. J. Physiol..

[105] Buntain H.M., Greer R.M., Wong J.C., Schluter P.J., Batch J., Lewindon P., Bell S.C., Wainwright C.E. (2005). Pubertal development and its influences on bone mineral density in Australian children and adolescents with cystic fibrosis. J. Paediatr. Child Health.

[106] Kelly A., Schall J.I., Stallings V.A., Zemel B.S. (2008). Deficits in bone mineral content in children and adolescents with cystic fibrosis are related to height deficits. J. Clin. Densitom..

[107] Bournez M., Bellis G., Huet F. (2012). Growth during puberty in cystic fibrosis: A retrospective evaluation of a French cohort. Arch. Dis. Child..

[108] Reiter E.O., Stern R.C., Root A.W. (1981). The reproductive endocrine system in cystic fibrosis. I. Basal gonadotropin and sex steroid levels. Am. J. Dis. Child..

[109] Allan J.L., Townley R.R., Phelan P.D. (1974). Family response to cystic fibrosis. Aust. Paediatr. J..

[110] Torres-Santiago L., Mericq V., Taboada M., Unanue N., Klein K.O., Singh R., Hossain J., Santen R.J., Ross J.L., Mauras N. (2013). Metabolic effects of oral versus transdermal 17β-estradiol (E_2_): A randomized clinical trial in girls with Turner syndrome. J. Clin. Endocrinol. Metab..

[111] Wei C., Crowne E.C. (2016). Recent advances in the understanding and management of delayed puberty. Arch. Dis. Child..

[112] Thaker V., Carter B., Putman M. (2021). Recombinant growth hormone therapy for cystic fibrosis in children and young adults. Cochrane Database Syst. Rev..

[113] Ahmad A., Ahmed A., Patrizio P. (2013). Cystic fibrosis and fertility. Curr. Opin. Obstet. Gynecol..

[114] Patrizio P., Zielenski J. (1996). Congenital absence of the vas deferens: A mild form of cystic fibrosis. Mol. Med. Today.

[115] Brugman S.M., Taussig L.M., Taussig L.M. (1984). The reproductive system. Cystic Fibrosis.

[116] Leifke E., Friemert M., Heilmann M., Puvogel N., Smaczny C., von zur Muhlen A., Brabant G. (2003). Sex steroids and body composition in men with cystic fibrosis. Eur. J. Endocrinol..

[117] Patrizio P., Salameh W.A. (1998). Expression of the cystic fibrosis transmembrane conductance regulator (CFTR) mRNA in normal and pathological adult human epididymis. J. Reprod. Fertil. Suppl..

[118] Bhasin S., Brito J.P., Cunningham G.R., Hayes F.J., Hodis H.N., Matsumoto A.M., Snyder P.J., Swerdloff R.S., Wu F.C., Yialamas M.A. (2018). Testosterone Therapy in Men With Hypogonadism: An Endocrine Society Clinical Practice Guideline. J. Clin. Endocrinol. Metab..

[119] Devuyst O., Golstein P.E., Sanches M.V., Piontek K., Wilson P.D., Guggino W.B., Dumont J.E., Beauwens R. (1997). Expression of CFTR in human and bovine thyroid epithelium. Am. J. Physiol..

[120] Li H., Ganta S., Fong P. (2010). Altered ion transport by thyroid epithelia from CFTR(-/-) pigs suggests mechanisms for hypothyroidism in cystic fibrosis. Exp. Physiol..

[121] Dolan T.F., Gibson L.E. (1971). Complications of iodide therapy in patients with cystic fibrosis. J. Pediatr..

[122] Azizi F., Bentley D., Vagenakis A., Portnay G., Bush J.E., Shwachman H., Ingbar S.H., Braverman L.E. (1974). Abnormal thyroid function and response to iodides in patients with cystic fibrosis. Trans. Assoc. Am. Physicians.

[123] Zimmermann M.B., Köhrle J. (2002). The impact of iron and selenium deficiencies on iodine and thyroid metabolism: Biochemistry and relevance to public health. Thyroid.

[124] Kauf E., Dawczynski H., Jahreis G., Janitzky E., Winnefeld K. (1994). Sodium selenite therapy and thyroid-hormone status in cystic fibrosis and congenital hypothyroidism. Biol. Trace Elem. Res..

[125] Segall-Blank M., Vagenakis A.G., Shwachman H., Ingbar S.H., Braverman L.E. (1981). Thyroid gland function and pituitary TSH reserve in patients with cystic fibrosis. J. Pediatr..

[126] De Luca F., Trimarchi F., Sferlazzas C., Benvenga S., Costante G., Mami C., Di Pasquale G., Magazzu G. (1982). Thyroid function in children with cystic fibrosis. Eur. J. Pediatr..

[127] Volta C., Street M.E., Ziveri M.A., Bonelli P., Spaggiari C., Grzincich G.L., Bernasconi S. (2005). Thyroid function, cytokine and IGF-IGFBP interactions in cystic fibrosis patients. Horm. Res..

[128] Naehrlich L., Dörr H.G., Bagheri-Behrouzi A., Rauh M. (2013). Iodine deficiency and subclinical hypothyroidism are common in cystic fibrosis patients. J. Trace Elem. Med. Biol..

[129] Lee S.Y., Chesdachai S., Lee M.J., He X.M., Tangpricha V., Braverman L.E. (2016). Thyroid Function in Patients with Cystic Fibrosis: No Longer a Concern?. Thyroid.

